# Sex- and age- dependent effect of pre-gestational chronic stress and mirtazapine treatment on neurobehavioral development of Wistar rat offspring

**DOI:** 10.1371/journal.pone.0255546

**Published:** 2022-02-03

**Authors:** Mireia Viñas-Noguera, Kristína Csatlósová, Eva Šimončičová, Ezster Bögi, Eduard Ujházy, Michal Dubovický, Kristína Belovičová

**Affiliations:** 1 Centre of Experimental Medicine, Institute of Experimental Pharmacology and Toxicology, Slovak Academy of Sciences, Bratislava, Slovakia; 2 Faculty of Natural Sciences, Comenius University, Bratislava, Slovakia; 3 Jessenius Faculty of Medicine, Comenius University, Martin, Slovakia; 4 Division of Medical Sciences, University of Victoria, Victoria, BC, Canada; Federal University of Santa Catarina, BRAZIL

## Abstract

Hormonal fluctuations, such as the perinatal period, may increase susceptibility of women to depression, which in turn exert a negative impact on child’s neurodevelopment, becoming a risk factor in development of neuropsychiatric disorders. Moreover, the use of antidepressants during this critical period presents a serious health concern for both the mother and the child, due to the consequences of treatment in terms of the reliability and safety for the proper neurodevelopment of the organism being not well known. Atypical antidepressants, such as mirtazapine, that targets both serotonergic and noradrenergic systems in the central nervous system (CNS), represent a novel focus of research due to its unique pharmacological profile. The aim of this work was to study the effects of maternal depression and/or perinatal antidepressant mirtazapine treatment on the neurobehavioral development of the offspring. Pre-gestationally chronically stressed or non-stressed Wistar rat dams were treated with either mirtazapine (10 mg/kg/day) or vehicle during pregnancy and lactation followed by analysis of offspring’s behavior at juvenile and adolescent age. We found mirtazapine induced significant alterations of nursing behavior. In offspring, pregestational stress (PS) had an anxiogenic effect on adolescent males (p≤0.05) and increased their active behavior in forced swim test (p≤0.01). Interaction between pregestational stress and mirtazapine treatment variously induced anxiolytic changes of juvenile (p≤0.05) and adolescent (p≤0.05) females and impairment of spatial memory (p≤0.01) in adolescent females as well. Hippocampal density of synaptophysin, pre-synaptic protein marker, was decreased mainly by mirtazapine treatment. In conclusion, our results show mirtazapine induced significant alterations in maternal behavior and several sex- and age-dependent changes in neurobehavioral development of offspring caused by both prenatal mirtazapine treatment and/or chronic pregestational stress.

## Introduction

An estimation of 17% men and 25% women experience an episode of major depressive disorder (MDD) at least once in their life [1]. Higher susceptibility of women to depression may arise from the increased vulnerability caused by periods of hormonal fluctuation, such as the perinatal period [2, 3].

The hormonal changes that take place during pregnancy expose the mothers to environmental challenges and have a clear impact on behavior. Steroid hormones play a crucial role in maternal mood both during and after gestation [4, 5]. However, the influence of specific hormones is different at these two time points. During pregnancy, production of estrogen and progesterone by placenta increase exponentially after week 6, and immediately after delivery the levels of these two hormones suddenly drop, until reaching pre-pregnancy levels [6, 7]. Studies in rodents show the importance of estrogen for fetoplacental nutrient transport, as well as its mood-elevating and antidepressant properties, as estrogens unite with the serotonergic system by increasing serotonin synthesis and decreasing its degradation [5]. Progesterone stimulates maternal food intake and is responsible for adequate accommodation of the uterus to the baby and to prevent pre-term birth. It also inhibits lactation until the baby is born and avoids fetus rejection caused by maternal cell-mediated immunity [8]. Its effects on mood may be also related to its binding to neurotransmitter membrane receptors, specifically serotonin. Keeping the right balance of these two hormones are very important for optimal welfare. Too low level of progesterone and domination of estrogen can lead to mental instability related to low mood [7, 8]. The two other important hormones that have been highly correlated with mood alterations during pregnancy are dehydroepiandrosterone (DHEA) and testosterone. After delivery, testosterone levels rise and become the most important hormone associated with negative mood, with a high correlation index, suggesting its severe role in the etiology of post-partum depression [9], while higher levels of DHEA during pregnancy were associated with better mood but after delivery its decline was related to mood aggravation. Nevertheless, it is not the effect of solely one of them but the combined action of different hormones and neurotransmitters at different time points that induce mood alterations.

Moreover, the rise on progesterone and prolactin and their effect in neuropeptide Y may explain increased maternal food intake, that accumulates as white fat and is used for both mother and fetus as energy source. Fetal and neonatal development is sustained by the uteroplacental blood flow and milk production after delivery, but a discoordination of hormonal production from placenta and pituitary gland, caused by maternal malnutrition, hypertension, smoking or preeclampsia among others, may alter fetal and neonatal demands [6].

Apart from the vulnerability of pregnant women to physiological alterations due to hormonal fluctuations, psychological challenges have been a topic of debate for clinicians and researchers. Pregnant women that express depressive symptoms are at higher risk of inflammatory and stress-related response, which associate with suboptimal features in the newborns. Treatment of stress-related disorders such depression during gestation rises a number of concerns related to the safety of these drugs for the developing fetus. However, the discontinuation of treatment may predispose the fetus to developmental complications later in life, making it difficult to discern the best approach in terms of reduced consequences for both the mother and the infant. Newborn children from depressed mothers tend to have preterm delivery, low birth weight and Apgar scores, as well as tendency to present a dysregulated HPA axis, that later in life manifest as poor social interactions, more negative attitude and altered emotional circuitry [10].

Second-generation antidepressants (SGA), such as selective-serotonin reuptake inhibitors (SSRIs) (fluoxetine, sertraline) or serotonin-norepinephrine reuptake inhibitors (SNRIs) (venlafaxine) introduced in Europe in the 1980s, are recorded as the first line antidepressant treatment during pregnancy by most medication guides [11, 12]. The World Health Organization (WHO) estimates that depression is a leading cause of disability worldwide [13]. However, the main concern for specialists is the impact of the treatment on the developing fetus/child, namely risk of potential congenital malformations, neonatal withdrawal symptoms or poor neonatal adaptation syndrome as well as long-term neurodevelopmental consequences [14–17]. In addition, the delay in treatment efficacy and the presence of many side effects led researchers to investigate alterative antidepressants with better efficacy, faster onset of action and lesser counterproductive reactions. Tricyclic antidepressants (TCAs), released in 1959, were commonly prescribed for the treatment of Major Depressive Disorder, but the adverse effects provoked by their anticholinergic activity such tachycardia, hypertonia and irritability, promoted its replacement for the treatment of this pathology by SSRIs [18]. Apart from SSRIs and SNRIs, atypical antidepressants such bupropion and mirtazapine are nowadays considered as alternative treatments. However, more research is needed to elucidate the mechanisms of action of these compounds. Bupropion has been linked to cardiac defects and increased risk of abortion. Mirtazapine, beneficial in terms of absence of cholinergic, adrenergic and serotonergic side effects caused by other antidepressants, as well as good tolerability and faster mitigation of symptoms, may predispose to gestational diabetes [10, 19].

**Fig 1:**
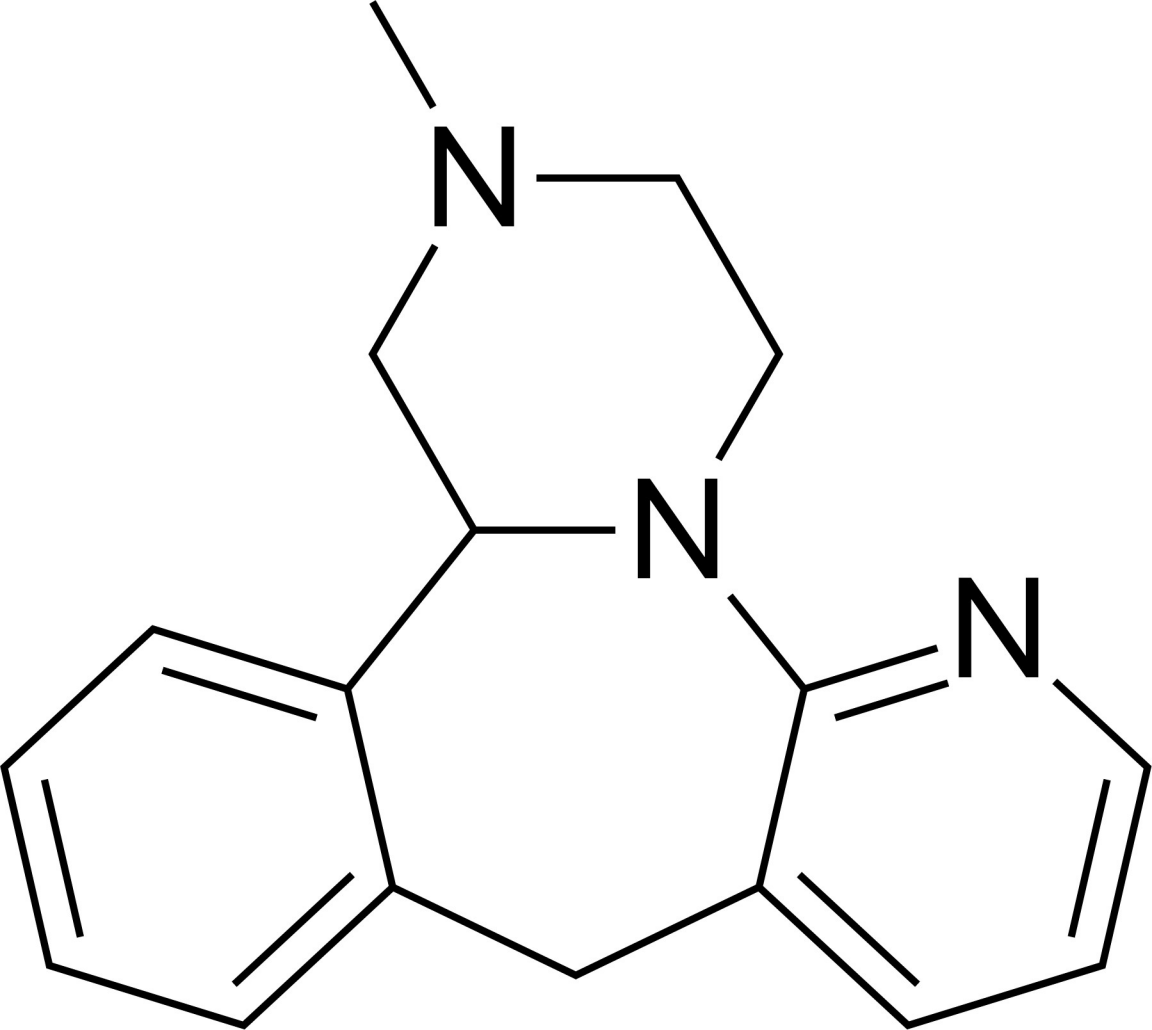
Chemical structure of mirtazapine.

Mirtazapine (MIR) (Fig 1) has a tetra-cyclic chemical structure with molecular weight of 265.36 and belongs to the piperazino-azepine group of compounds. It is a new generation antidepressant that has a different mechanism of action than SGAs, targeting both the serotonergic and noradrenergic systems in the CNS. It is a noradrenaline (NE) and specific serotonin (5-HT) antidepressant (NaSSA) that acts as an antagonist at central α2-adrenergic inhibitory autoreceptors and heteroreceptors, as well as at the 5-HT_2_ and 5-HT_3_ receptors. MIR enhances the release and availability of NE by blocking presynaptic inhibitory α2-autoreceptors and enhances the 5-HT release by antagonism of the α2-heteroreceptors in the serotonergic nerve terminals and simultaneously blockade of postsynaptic 5-HT_2_ and 5-HT_3_ receptors. Thanks to this double activity, MIR is suggested to induce earlier onset of antidepressant effects avoiding the serotonergic related side effects such as high body temperature, agitation, increased reflexes, tremor, sweating, dilated pupils, and diarrhea [20]. However, it may induce an enhanced body weight gain and sleepiness [21, 22]. Mirtazapine has low *in vitro* affinity for central and peripheral dopaminergic, cholinergic, and muscarinic receptors, but high affinity for central and peripheral histamine H1 receptors. However, it appears that the antihistaminergic effects of the drug are counteracted by noradrenergic transmission when the drug is commenced at dosages ≥15 mg/day, i.e. within the recommended dosage range [23]. Initial dose of MIR is 15 mg/day orally once a day at bedtime and maintenance dose represents 15 to 45 mg orally once a day [24]. Even though MIR seems to represent a plausible alternative of antidepressant medication during gestation and there is no evidence that use of mirtazapine in pregnancy causes birth defects, preterm birth, or low infant birth weight. While the evidence for other pregnancy outcomes is also reassuring, only small numbers of women have been studied and number of animal studies is not sufficient [25].

To date, few studies have been reported to analyze the excretion of antidepressant mirtazapine (by means of milk/plasma (M/P) ratio) into breast milk. Aichhorn and colleagues reported that mirtazapine was excreted in breast milk in a nursing mother suffering from postpartum depression. Mirtazapine concentration in foremilk was similar to maternal plasma, ranging between 7 and 34 ng/mL, but concentration in hindmilk was higher than in mother’s plasma. Finally, they postulated that the serum concentration in the infant (0.2 ng/mL) was below the therapeutic range for adults (30 to 80 ng/mL) [26]. Klier research team studied the mirtazapine levels in breastmilk at the expected time of peak concentration, assuming that breastmilk levels peak at similar timepoints as serum levels. They found that mirtazapine is excreted into breastmilk, but infant exposure is minimal, between 0.21–1.02% of the weight-adjusted maternal dose [27]. Kristensen and colleagues studied eight breast-feeding woman who were being treated with mirtazapine pre- or postnatally. The milk transfer of mirtazapine was moderate, with a M/P ratio of 1.1, and no drug-related adverse effects were detected. They concluded that short-term use of mirtazapine during breastfeeding is secure [28]. Finally, Tonn research team observed a 1.3% child absorption of mother’s daily mirtazapine, with a 3.3 reported serum levels in infant, almost within the adult therapeutic range, that compared to the 0.12 in mother’s, postulated a less efficient elimination by child metabolism [29]. Quantification of antidepressant concentration in different body fluids such as milk may be a useful indication of fetal exposition to mother treatment and its further implications for the child. In addition, the transfer amount of each specific compound may vary according to its pharmacological properties such as protein binding, lipid content of milk and drug concentration on mother serum [30, 31].

Despite lacking efficient translatability, several animal models, including chronic unpredictable stress, have been investigated for decades to evaluate depression-like behavioral changes and their potential mechanisms of action. Studies in rodents show that chronic pre-gestational and prenatal maternal stress, which disrupt the maternal endocrine, nervous and immune systems, can induce long-term alterations in the synaptic structure and so impact the behavioral outcomes in the offspring [32, 33]. Apart from the regulatory roles of serotonin, norepinephrine and dopamine neurotransmitters in adult brain, monoamines play an important role in the fetal maturation of the brain, such as—during neuronal proliferation, migration and differentiation, myelinization and synaptogenesis [34]. The exposure to stressful situations early in life may disable the optimal structural and functional development of hippocampus, due to the damaging action of excessive corticosterone concentration and dysregulation of monoamines [15, 35]. Persisting high levels of stress are thought to result in loss of synapses in circuits underlying affective and cognitive processes. These reductions are presumed to contribute to the symptoms of depression associated with major depressive disorder [36]. Synaptophysin is a synaptic vesicle glycoprotein, which immunoreactivity is present in a punctate pattern in the hippocampus and has been used as a presynaptic marker for quantification of synapses [37]. The hippocampal formation consists of several histologically distinguishable modules, such as cornu ammonis (CA) regions (CA1, CA2, CA3, CA4), dentate gyrus (DG), presubiculum, and subiculum. These regions of the hippocampus are associated with different functions (e.g. memory encoding and retrieval) and may be specifically disrupted in various diseases [38]. Granule cells, the main output cells of the DG, send axons (mossy fibers) through CA4 to CA3 and innervate a small number of pyramidal cells and a disproportionally large number of interneurons CA3 pyramidal cells then form recurrent excitatory network and send axons to CA1. Theoretical work, as well as anatomical, physiological, and behavioral experiments support the idea that the DG-CA3 system performs the pattern separation and the pattern completion of the inputs to the hippocampus, operations needed for memory encoding and retrieval [39]. Purpose of the hippocampus happens to be severely affected by early life stress, predisposing the individual to an impaired reactivity when exposed to adverse environmental stimuli [40]. In our previous study, we have shown that pre-gestational maternal stress may affect hippocampus at the time of birth by increasing the resting membrane potential, suppressing depolarization-activated action potential firing, and increasing the spontaneous activity of hippocampal cells from newborn rat offspring [41], but pre-gestational stress induced changes in different subregions of hippocampus are not thoroughly known. However, antidepressant treatment may facilitate the challenged neurogenesis by, upregulating the hippocampal concentration of glucocorticoid receptors, which help to attenuate the hyperactivity of the HPA axis and inducing morphological changes in the neuronal network [15, 40, 42]. Some studies suggest that developmental fluoxetine (SSRI) exposure of prenatally stressed male and female offspring reversed the effect of stress on the number of immature neurons in the dentate gyrus (DG), with effects being more prevalent in adult male offspring [43, 44]. However, knowledge of mirtazapine treatment’s impact on pregnancy and lactation hippocampal neurogenesis of juvenile, adolescent and adult offspring during last days of pregnancy and for following 2 weeks postpartum (PP), is very limited.

The aim of the present study was to determine the possible implications that pre-gestational chronic stress and administration of the new generation antidepressant mirtazapine have on the behavioral and neurodevelopmental outputs in the offspring of both sexes. Aiming our focus on functional brain developments that starts on day 10 PP and continues through juvenile and adolescent age [45–48].

## Materials and methods

### Animal breeding

Female nulliparous Wistar rats (initial weight 200–220 g, age 2–3 months, n = 44) used in this study were obtained from the Department of Toxicology and Laboratory Animal Breeding Station of the Institute of Experimental Pharmacology and Toxicology, Centre of Experimental Medicine of the Slovak Academy of Sciences, Dobra Voda, Slovak Republic. After 7 days of acclimatization, females were randomly assigned to stress or non-stress groups. Animals in the stress group were exposed to unpredictable stressors of mild intensity for a total of 3 weeks. One week after the end of the stress procedure, females were mated with males in the ratio 3:1. The presence of spermatozoa in vaginal smears was considered day 0 of gestation. On day 15 of gestation, the females were separated and housed individually. This may cause some level of stress; however, it is necessary due to ongoing pregnancy and all animals underwent the same process. The animals had *ad libitum* access to food pellets and water and were kept in a temperature and humidity-controlled room (20–24°C and relative humidity 50–60%) with 12/12 hours of light/dark cycle. The experiments were conducted in compliance with the Principles of Laboratory Animal Care issued by the Ethical Committee of the Institute of Experimental Pharmacology and Toxicology, Slovak Academy of Sciences, Bratislava, and the experimental design was approved by the State Veterinary and Food Administration of the Slovak Republic.

One day after birth, litters were culled to four males and four females (with eight offspring per cage). Reproductive variables were recorded right after the birth. The offspring were weaned on post-partum day 21 and housed in litter groups of same-sex four animals per cage. No more than two pups from the same mother per group (n = 6–8 animals/group) were used for behavioral testing.

### Chronic unpredictable stress schedule

**Fig 2:**
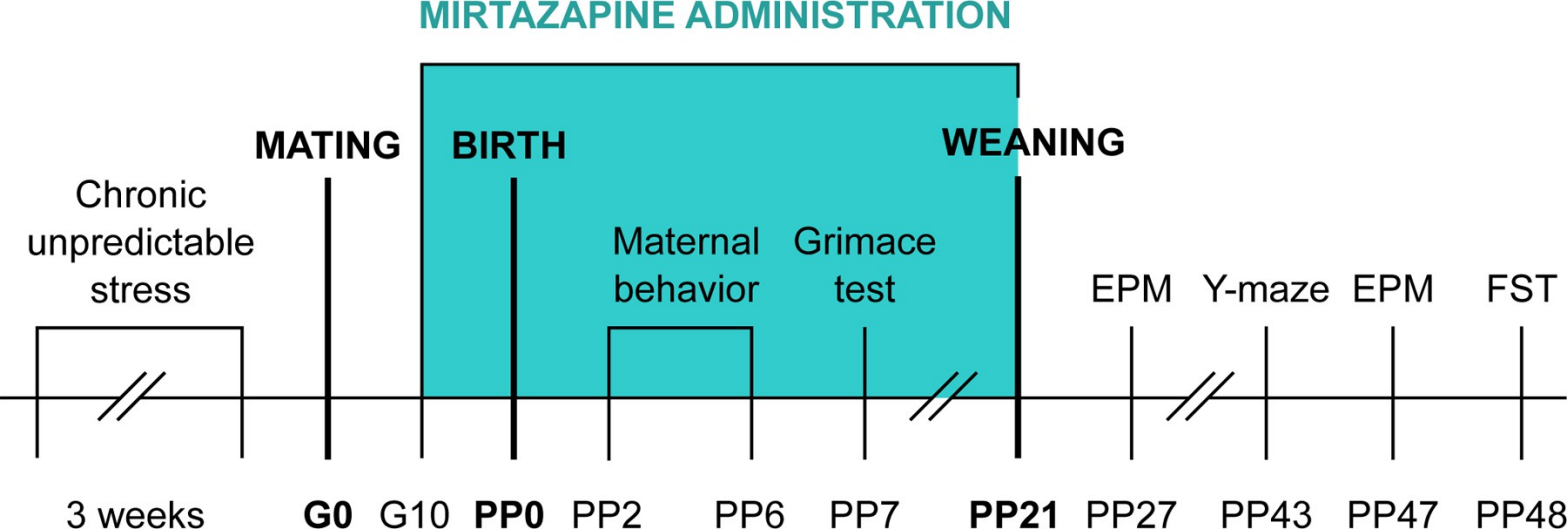
Schedule of the experiment. G- gestation day; PP-post-partum day; EPM- elevated plus maze; FST- forced swim test.

In order to not expose females to stressors during pregnancy but rather study ongoing effects of chronic stress, females were assigned to stress or non-stress groups prior to breeding (Fig 2). Animals from non-stress group were left in their cages in separate room with standard room conditions mentioned above. The animals were exposed to 1–2 stressors per day. The list of stressors included: 1) Overcrowding—6 rats housed together (24h); 2) Exposure to damp bedding (12h); 3) Food deprivation (24h); 4) Predator stress–cloth with cat odor (10h); 5) Water deprivation (12h); 6) Cage decline at 45-degree angle (6h); 7) Strobe light- flicker lights (12h).

### Mirtazapine treatment

Mirtazapine (Mikrochem Trade spol. s r.o.) with a molecular weight of 265.36 was diluted in citric acid and administered orally via the wafer biscuit (size of 1 cm^3^) to pregnant rats from day 10 of gestation until sacrifice at the clinically relevant dose of 10 mg/kg once per day. Pups were receiving mirtazapine via mother until weaning on day 21 *post-partum* (PP). This dose was chosen based on a body surface area normalization (BSA) conversion used to determine the starting human dose extrapolated from animal studies. The Km factor, which is the ratio of body weight (kg) to body surface area (m^2^), is used to convert doses expressed in mg/kg to units of mg/m^2^ [49]. Dose 10 mg/kg a day was used to simulate low dose end in humans. The dams from control groups received 1 cm^3^ of wafer biscuit once a day filled with vehicle (water). Feeding was completed under investigator’s supervision to ensure the dam consumed the entire biscuit. Pregnant dams were randomly divided into 4 groups: non-stress + VEHIC (vehicle), non-stress + MIR, stress + VEHIC, stress + MIR.

### Rat Grimace Scale

Mothers (n = 6–7 animals/group) were placed in individual cages for 10 min and each minute 4 pictures were taken from each rat from front with a Nikon professional camera. Animals were tested 5 weeks after CUS. Each picture was evaluated by a score from 0 to 2 according to the following four action units [50]:

1. Orbital tightening: rats in pain display narrowing of the orbital area which manifests as partial or complete eye closure or squeezing.
2. Nose/cheek flattening: less bulging of nose and cheek and absence of crease between cheek and whisker pads.
3. Ear changes: ears in pain tend to fold, curl and angle forwards or outwards, and the space between ears is wider.
4. Whisker change: whiskers move forward (away from face) and tend to bunch.

### Behavioral tests

All tests, except maternal behavior, were carried out between 8:00 a.m. and 12:00 p.m.

### Maternal behavior

Mothers (n = 6–10 animals/group) were observed for 5 consecutive days from PD2 until PD6 two times per day for 5 min based on previous literature [32]. Observations took place in the morning (between 8:30 a.m. and 10.00 a.m.) and the afternoon (between 2:30 p.m. and 3:30 p.m.). Time and number of bouts in the following maternal behaviors were recorded: licking; licking/nursing; nursing (arched-back nursing, blanket nursing, and/or passive nursing); nest building and time off the pups. Differences between groups in maternal behaviors were assessed using total time spent in these maternal behaviors.

### Forced swim test (FST)

The FST consists of a container filled with water where the animal is placed and cannot escape. The apparatus consists of a vertical cylindrical glass container (height 45 cm, diameter 25cm) filled with tap water at 23±1°C. The water volume is enough to ensure that the animals can not touch the bottom of the container with their hind paws. The forced swim test was conducted over two days. On the first day, rats were introduced to the cylindrical glass tank filled with water for 15 min (not videotaped), towel-dried and returned to their home cage. Twenty-four hours later, the animals (n = 7–11 animals/group/sex) were exposed to the same experimental conditions for 5 min, dried and returned to their home cage. Sessions were videotaped and scored using the software ANYMAZE™ (Stoelting Europa, Co., Ireland). The behavior scored in the forced swim test concerns: (1) immobility- floating with the absence of any movement, (2) latency to be immobile- time duration, (3) swimming, (4) climbing. Offspring was tested at the age of 48 days.

### Elevated plus maze (EPM)

All parts of the apparatus are made of dark polyvinyl plastic. The open and the closed arms of the maze are 50cm above the floor, 50cm long and 10cm wide. Two tests were running simultaneously. The movements of the rats were tracked with digital camera and the individual sessions were analyzed by computer software ANYMAZETM (Stoelting Europe, Ireland). Mild light was provided by a lamp attached above the open arms of the maze. Each session lasted 5 minutes and was started by placement of the rat in the central area facing the open arms of the maze. After each individual trial, the maze was wiped with a mild detergent. The behaviors scored were: (1) distance traveled in the open arms of the elevated plus maze, and (2) time spent in the open arms of the elevated plus maze. The animals were tested at the age of 27 (n = 7–8 animals/group/sex) and 47 days (n = 7–10 animals/group/sex).

### Y-Maze

The Y-Maze Test is widely used to assess exploratory behaviors, learning, and memory function in rodents and short-term memory [51]. The apparatus was made from black Plexiglas (50x16x32 cm) in which the arms were symmetrically separated at 120°. No visual cues were placed inside the maze, but different extra-maze cues were visible from all three arms to enable spatial orientation. During the first trial, each animal could freely explore two arms of the maze for 15 min and its behavior was recorded with a camera. Subsequently, animals were returned to the home cage for one minute and the maze was cleaned with 70% ethanol. The second trial lasted 5 minutes with animal having the access to all three arms. Sessions were videotaped and scored using the software ANYMAZE™ (Stoelting Europa, Co., Ireland). The animals were tested at the age of 43 days (n = 5–8 animals/group/sex). Spontaneous alternation behavior (the alternation percentage is calculated by dividing the number of alternations by number of possible triads x 100) is considered to reflect spatial working memory, while the total number of arm entries was considered to reflect spontaneous locomotor activity [52, 53].

### Immunohistochemistry assay

Collection of tissue from offspring were done at different ages of the animals: in juvenile age (PP31) (n = 5–8 animals/group/sex) and in adolescent age (PP50) (n = 5–8 animals/group/sex). The animals were killed by cervical dislocation according to European commission guidelines [54]. Brains were extracted (not perfused) and post-fixed with 4% paraformaldehyde for 24 h, cryoprotected in 30% sucrose/phosphate-buffered saline solution for up to 1 week, rapid frozen with liquid nitrogen and kept at −80°C. Before immunohistochemistry assay, brain tissue was sliced in 40 μm sections on a cryostat (Leica). Tissue sections were stored in antifreeze solution at −15°C. The Ca3, Ca4 and DG of the dorsal hippocampus were assessed for the presynaptic marker synaptophysin (Monoclonal Anti-Synaptophysin, Sigma) as described before [32]. Tissue was first treated with 0.6% H_2_O_2_ for 30 minutes at room temperature and then incubated with 5% Normal Goat Serum (NGS) (Lampire Biological Laboratories) in Tris-Buffered Saline Tween at room temperature for 30 min to decrease probability of non-specific antibody binding, followed by overnight incubation at 4°C in mouse anti-synaptophysin (1:200, Sigma Aldrich). Sections were then incubated at room temperature for 2 h in biotinylated goat anti-mouse (1:200, Vector Laboratories). Brain sections were further processed using the Avidin-Biotin Complex (ABC Elite kit; 1:1000; Vector laboratories) and DAB kit (Vector laboratories). Sections were mounted on Starfrost Advanced Adhesive for IHC (Bamed) dried, dehydrated and coverslipped with Permount (Fisher Scientific).

### Quantification

**Fig 3:**
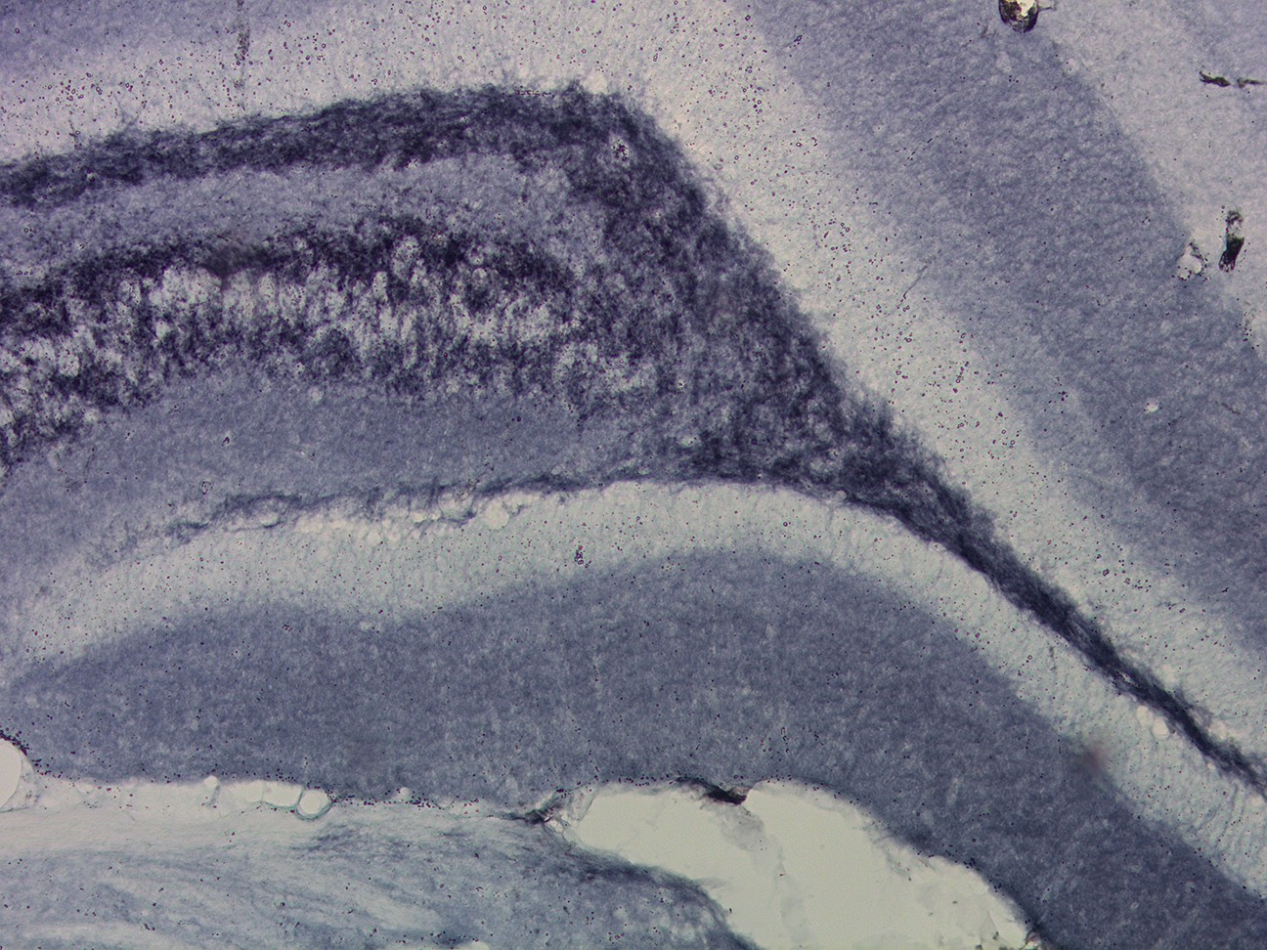
Immunohistochemical photograph. Representative picture of the synaptophysin stained dorsal hippocampus at 10 × magnification.

Sections of the dorsal hippocampus were analyzed for optical densities of synaptophysin (Fig 3). Immunoreactivity for all sections was examined under 40x objective using Leica DM4000M. Photomicrographs were taken for three areas within each analyzed hippocampal region, i.e., CA3, CA4 and DG. The software ImageJ64 (Wayne Rasband, NIH, Bethesda MD, USA) was used for assessment of optical densities for all immunoreactive cells. The relative optical density was defined as the difference in optical density (grey level) after calibration between the area of interest and the background, which was an equivalent area adjacent to the area of interest with minimal staining.

### Data analysis

Normality of data was analyzed by Shapiro-Wilks test, whereas data with p≤0.05 were considered not normally distributed. Data without normal distribution were analyzed using Kruskal-Wallis test (STATISTICA 10). Analysis of variance (factorial ANOVA) was used to evaluate differences in the individual variables of tests with normally distributed data (STATISTICA 10). Post-hoc comparisons utilized the Tukey HSD test. The data with normal distribution were expressed as mean ± standard error mean (S.E.M), data without normal distribution were expressed as box-and-whisker plot. The changes with values of p≤0.05 were considered statistically significant. All analysis was done in a blind manner.

## Results

### Mothers

#### Grimace test

**Fig 4:**
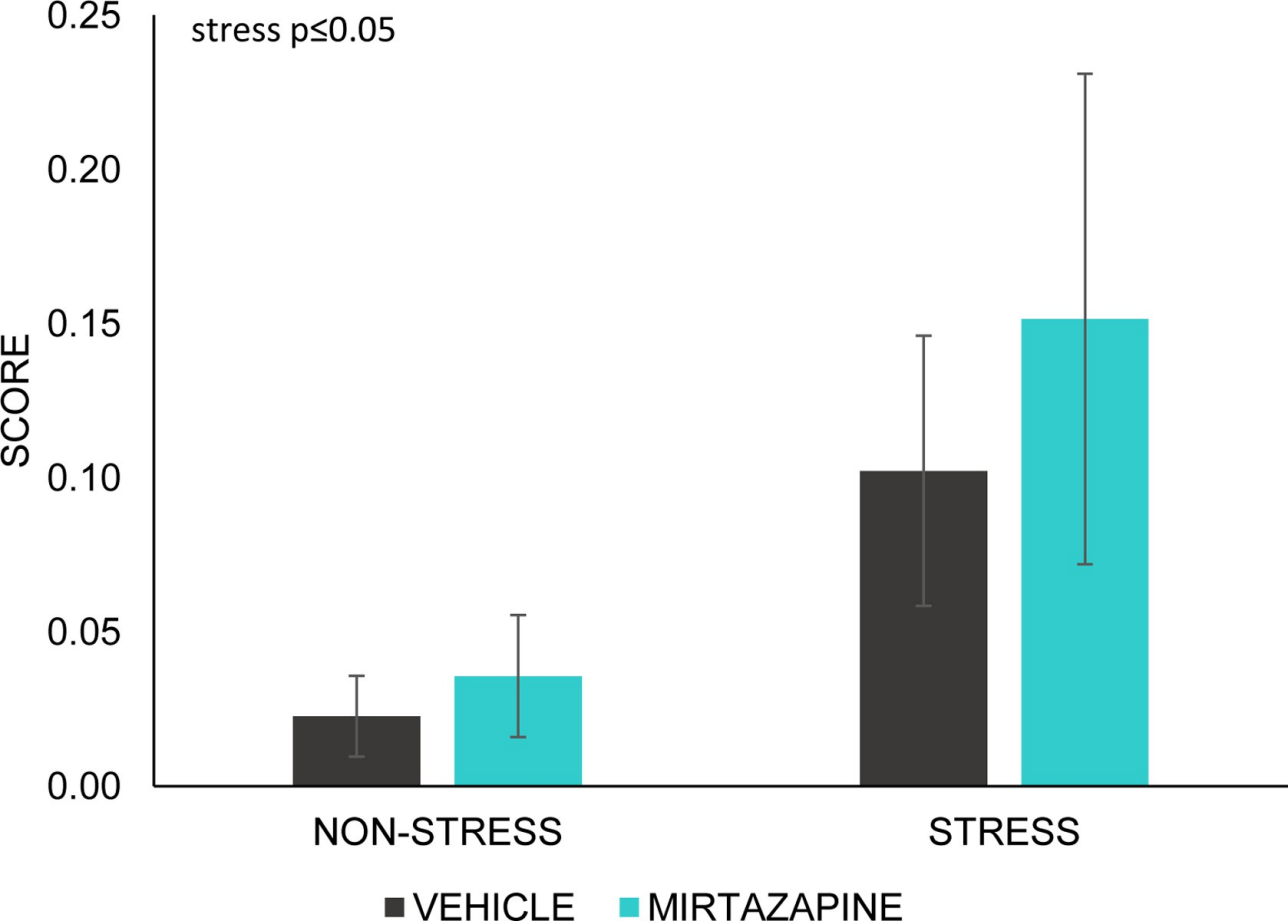
Grimace scale test. Data represent mean ± SEM. n = 6–7 animals/group.

Rat grimace test scores has been used to evaluate spontaneous pain [55]. We observed significant main effect of stress on grimace test scores (F (1, 22) = 5.00; p≤0.05). Post-hoc analysis did not reveal any significant differences (Fig 4).

#### Maternal behavior

**Fig 5:**
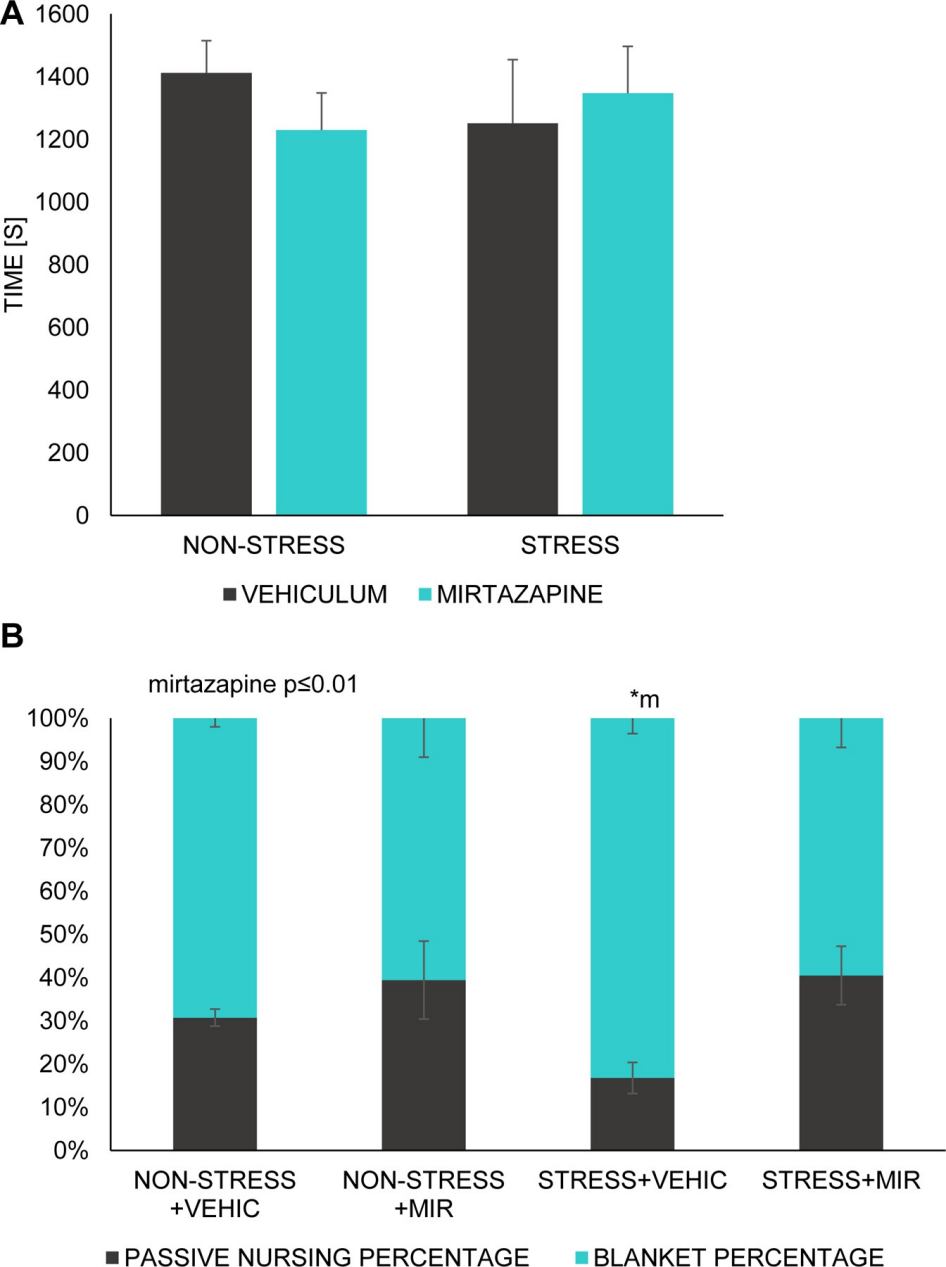
Maternal behavior. (A) total time spent nursing, (B) percentage of passive nursing and blanket nursing out of total time spent nursing. Data represent mean ± SEM. n = 6–10 animals/group. *p≤0.05; m-compared to both mirtazapine groups.

There was no significant main effect of stress or mirtazapine on total time dams spent nursing (Fig 5A), however, we observed significant main effect of mirtazapine on the percentage of passive nursing and blanket nursing (F (1, 30) = 10.74; p≤0.01). Subsequent post-hoc analysis showed decrease of passive nursing (p≤0.05) and increase of blanket nursing (p≤0.05) in stress × vehicle group as compared to both mirtazapine groups (Fig 5B).

### Offspring

#### Elevated plus maze- juvenile offspring

**Fig 6:**
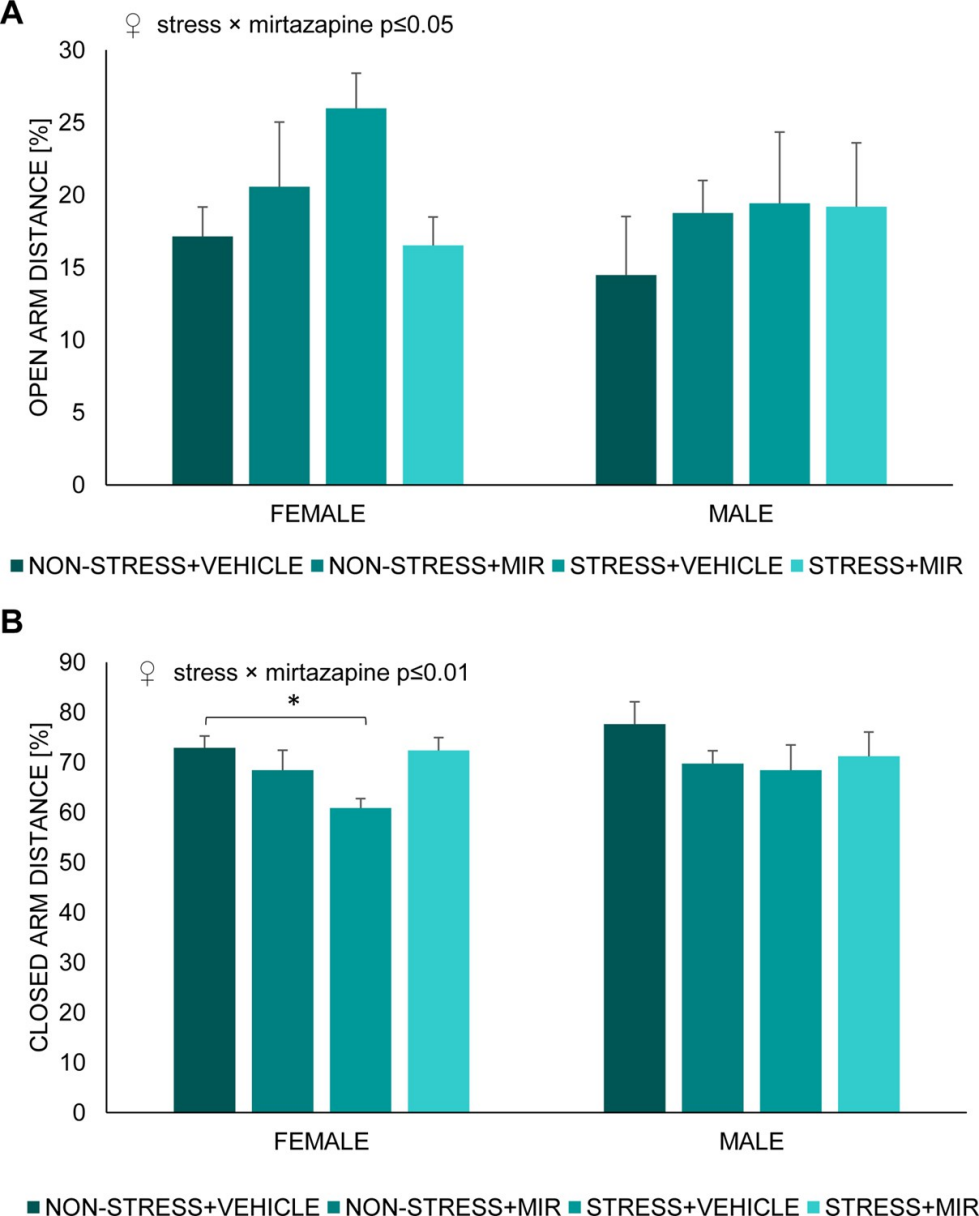
Percentage of distance travelled in individual arms of elevated plus maze by juvenile animals. (A) open arm distance, (B) closed arm distance. Data represent mean ± SEM. n = 7–8 animals/group/sex. MIR- mirtazapine; *p≤0.05.

We did not observe an effect of sex but there was a stress × mirtazapine interaction in females on percentage of distance travelled in the open (F (1, 24) = 4.89; p≤0.05) (Fig 6A) and closed arms (F (1, 24) = 7.82; p≤0.01) (Fig 6B) of EPM, an effect was not present in male offspring. Further analysis showed a decrease in total distance travelled in stress × vehicle females compared to control group.

#### Elevated plus maze- adolescent

Main effect of sex was present in all parameters of elevated plus maze with females being more active (higher total distance travelled) than males (F (1, 62) = 9.04; p≤0.01).

**Fig 7:**
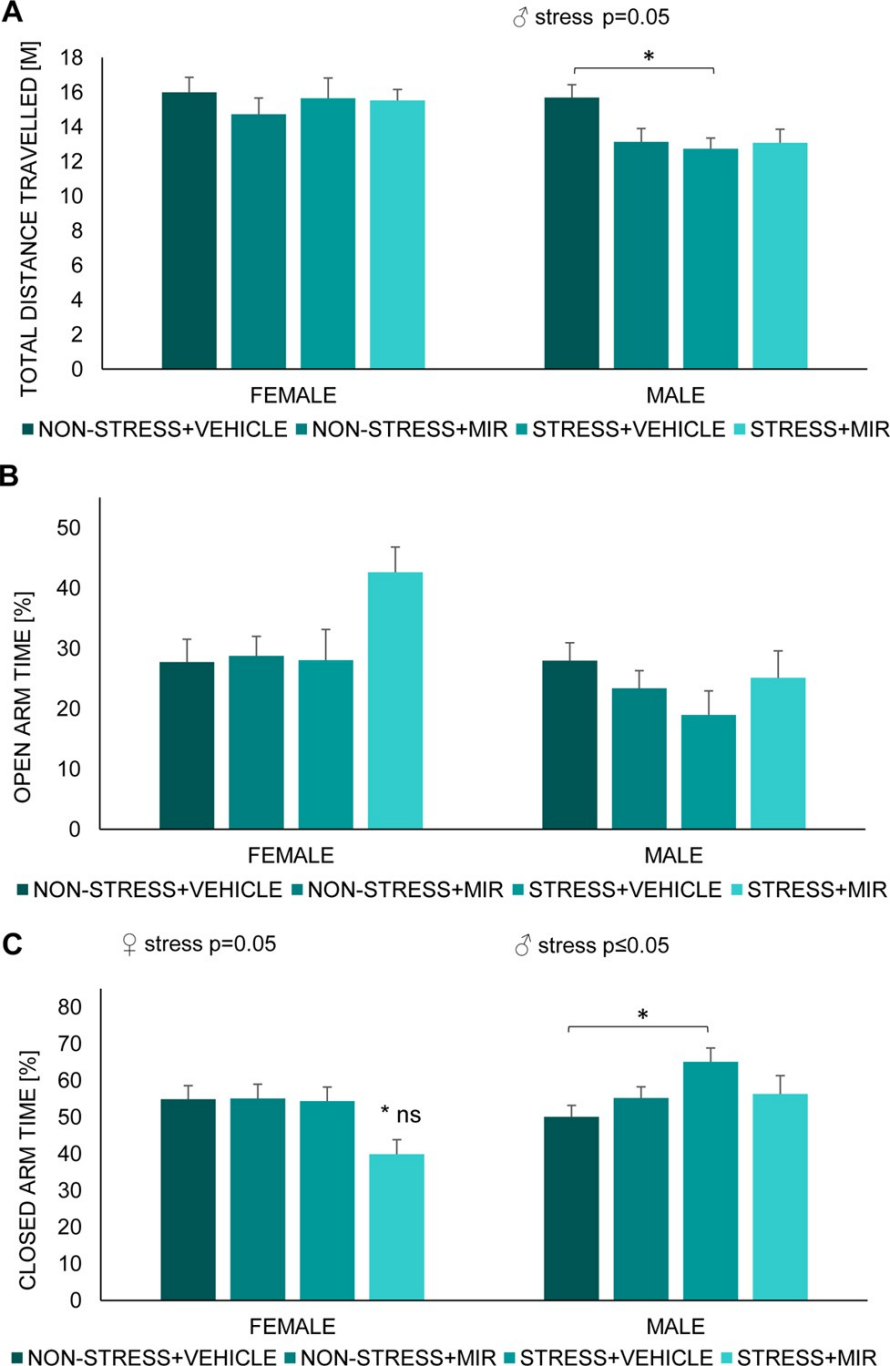
Elevated plus maze of adolescent offspring. (A) total distance travelled, (B) open arm time percentage, (C) closed arm percentage. Data represent mean ± SEM. n = 7–10 animals/group/sex. ns- compared to both non-stress groups; *p≤0.05.

In males, we observed significant main effect of stress (F (1, 30) = 3.93; p = 0.05) in total distance travelled. Further post hoc analysis showed significantly decreased activity in stress × vehicle group compared to non-stress × vehicle group (Fig 7A).

In males, there were no changes in the percentage of time spent in the open arm (Fig 7B) but we observed a main effect of stress (F (1, 30) = 4.53; p≤0.05) in the percentage of time spent in the closed arm. Post-hoc analysis revealed that animals from stress × vehicle group had significantly increased percentage of time spent in the closed arm compared to non-stress × vehicle group (p≤0.05) (Fig 7C).

In females, we did not observed changes in the percentage of time spent in the open arm (Fig 7B). however, there was significant main effect of stress (F (1, 32) = 4.08; p = 0.05) in the percentage of time spent in the closed arm. Post-hoc analysis showed decrease in stress × mirtazapine group significant compared to non-stress × vehicle (p≤0.05) and non-stress × mirtazapine group (p≤0.05) (Fig 7C).

#### Forced swim test

There were no sex differences in either of the selected forced swim test parameters.

**Fig 8:**
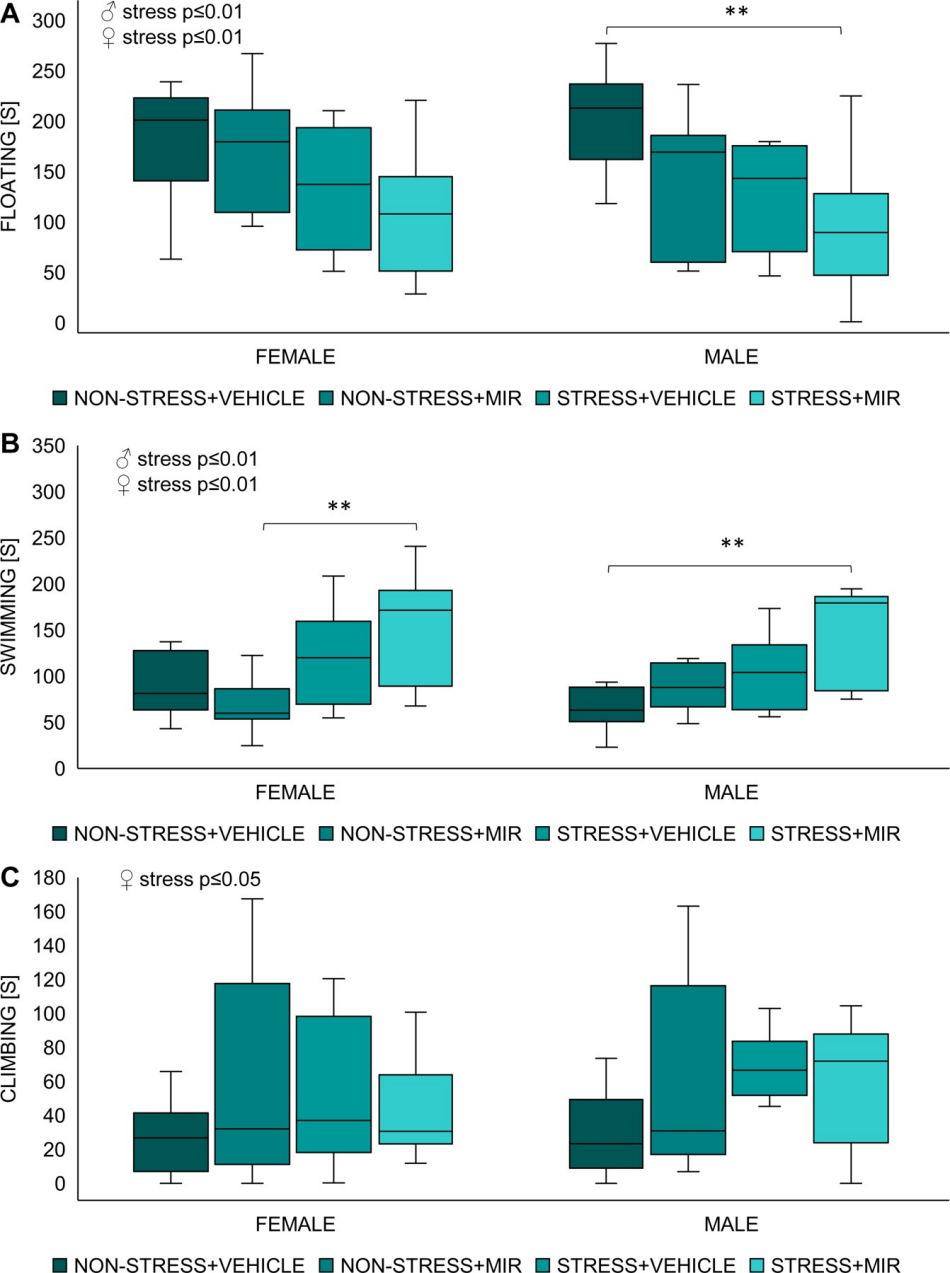
Forced swim test of adolescent offspring. (A) time spent floating, (B) time spent swimming, (C) time spent climbing. n = 7–11 animals/group/sex. **p≤0.01.

Kruskal-Wallis test showed significant main effect of stress in time spent floating (males: H = 7.41; p≤0.01; females: H = 6.90; p≤0.01), time spent climbing (males: H = 4.47; p≤0.05; females: not significant) and time spent swimming (males: H = 6.49; p≤0.01; females: H = 10.24; p≤0.001). Further analysis revealed that floating time of stress × mirtazapine males was significantly decreased compared to non-stress × vehicle males (p≤0.01), however floating time of females was not significantly changed between individual groups (Fig 8A). Total swimming time of stress × mirtazapine males was significantly decreased compared to non-stress × vehicle males (p≤0.01) and floating time of stress × mirtazapine females was significantly decreased compared to non-stress × mirtazapine females (p≤0.01) (Fig 8B). Climbing time was not significantly changed between individual groups in either sex (Fig 8C).

#### Y-maze- adolescent

We observed main effect of sex in both motor activity (F (1, 45) = 11.53; p≤0.01) as well as in percentage of spontaneous alterations (F (1, 45) = 4.27; p≤0.05) with females being more active but having decreased percentage of spontaneous alterations compared to males.

**Fig 9:**
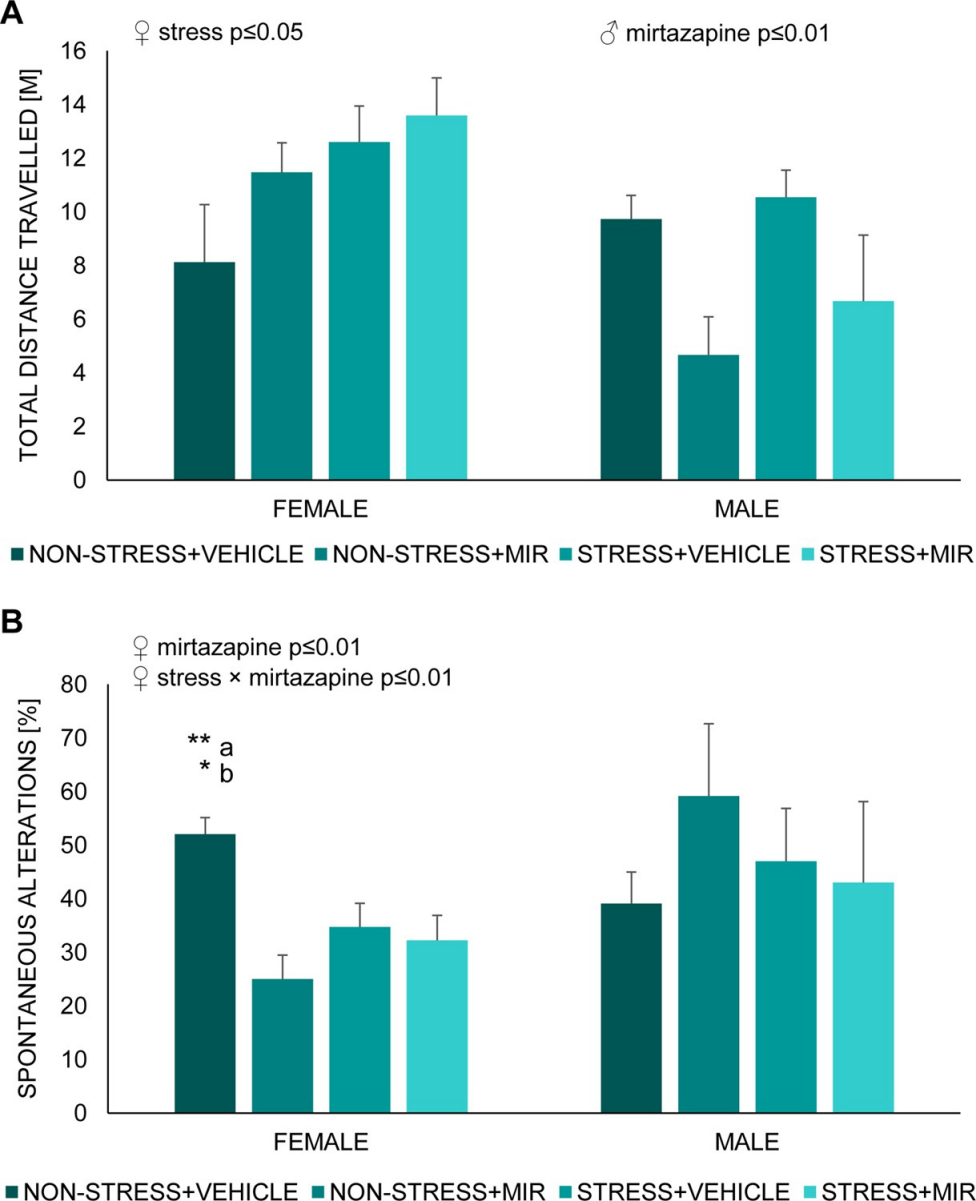
Y-maze of adolescent offspring. (A) total distance travelled, (B) percentage of spontaneous alterations. Data represent mean ± SEM. n = 5–8 animals/group/sex. a- compared to non-stress × mirtazapine, b- compared to stress × vehicle, *p≤0.05, **p≤0.01.

In males, a significant main effect of treatment (F (1, 20) = 9.79; p≤0.01) was observed in in total distance travelled. Post-hoc analysis revealed significant decrease in non-stress × mirtazapine group compared to stress × vehicle group (p≤0.05) (Fig 9A). We did not observe any significant changes in percentage of spontaneous alterations (Fig 9B).

In females, a significant main effect of stress was in total distance travelled (F (1, 25) = 4.95; p≤0.05), however post hoc analysis did not revealed individual changes between groups (Fig 9A). Further, we observed significant main effect of mirtazapine (F (1, 25) = 11.25; p≤0.01) and mirtazapine × stress interaction (F (1, 25) = 7.81; p≤0.01) in percentage of spontaneous alterations. Post-hoc analysis revealed significant decrease in both mirtazapine groups (non-stress × mirtazapine p≤0.01; stress × mirtazapine p≤0.05) compared to non-stress x vehicle group (Fig 9B).

#### Synaptophysin-juvenile

We did not observe any statistically significant changes in the synaptophysin optical density in the hippocampal areas Ca3, Ca4 and dentate gyrus.

#### Synaptophysin-adolescent

**Fig 10:**
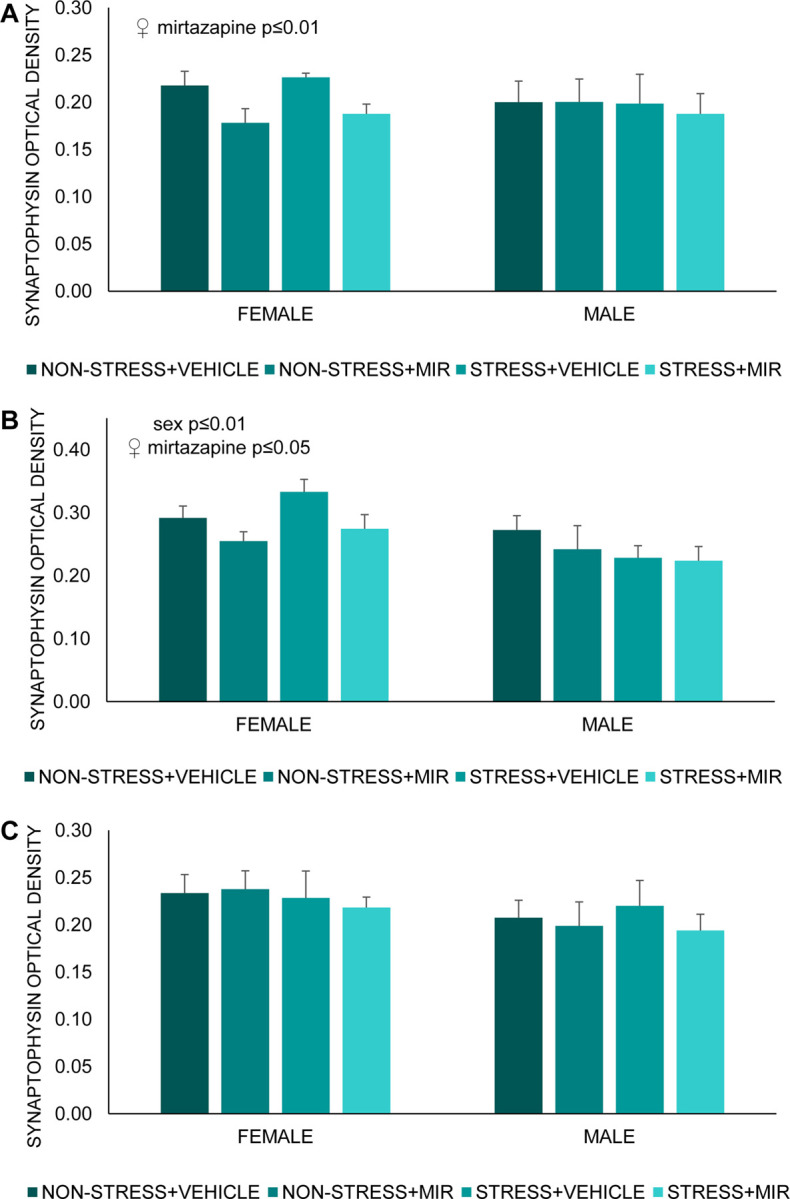
Optical density of synaptophysin in hippocampus. (A) Ca3 area, (B) Ca4 area, (C) dentate gyrus area. Data represent mean ± SEM. n = 5–8.

We found significant main effect of sex present only in area Ca4 of hippocampus (F (1, 47) = 7.23; p≤0.01) (Fig 10A). Further, we observed significant main effect of mirtazapine in hippocampal areas Ca3 (F (1, 19) = 9.01; p≤0.01) (Fig 10B) and Ca4 (F (1, 23) = 5.87; p≤0.05) (Fig 10A) of females. Post-hoc analysis did not reveal significant differences between groups. We did not find any changes in dentate gyrus (Fig 10C).

## Discussion

In the present study, focused on possible implications of pre-gestational chronic stress on the neurodevelopmental and behavioral outputs in the offspring of both sexes during juvenile age and adolescence, we investigated the consequences of administration of the atypical antidepressant mirtazapine. The results of our study showed that pre-gestational CUS and mirtazapine treatment can affect the behavior of the mothers. In adolescent offspring, CUS induced an increased active behavior, reflected in a more active swimming. In males, MIR treatment resulted in an increased anxiety-like behavior and a decrease in general motor activity. On the contrary, in females, CUS resulted in an increase in motor activity that MIR treatment also enhanced in the non-stressed group. In addition, in females, mirtazapine treatment during gestation and lactation had a long-term impact on the structural morphology of hippocampus synaptic markers, which may contribute to an impaired cognitive function in these animals. We evaluated the spontaneous pain of the mothers by the Rat Grimace Scale (RGS) and we found increased facial expression indicating pain in those mothers, which were exposed to stress schedule. RGS was developed to identify acute and inflammatory pain but it can be applied to a wider range of pain types and chronicity [56, 57] and this is supported by our data that suggest ongoing influence of CUS even 5 weeks after the CUS procedure.

Maternal behavior usually emerges close to parturition and after birth, with the female showing a very rapid interest in the new-born [58]. Detrimental changes in maternal behavior can generate permanent neurological and social inclusion disturbances in offspring [59]. We observed an effect of mirtazapine treatment on the proportion of passive to blanket nursing favoring the latter one, indicating a slight influence of mirtazapine on some aspects of maternal behavior. Mirtazapine acts by antagonizing the adrenergic alpha2-autoreceptors and alpha2-heteroreceptors as well as by blocking 5-HT2 and 5-HT3 receptors. It enhances, therefore, the release of norepinephrine and 5-HT1A-mediated serotonergic transmission [21]. Since maternal behavior is strongly regulated by serotonin [60–62], we thus suppose that serotonin activity of MIR can underlie these changes. Shift in maternal behavior in favor of more active blanket nursing caused by antidepressant treatment is in line with our previous results from venlafaxine (serotonin-norepinephrine reuptake inhibitor) [63] as well as results from fluoxetine (selective serotonin reuptake inhibitor) [64].

### Anxiety-like behavior

Offspring of mothers that have experienced stress during gestation may have impaired emotional development due to a dysregulation of the HPA axis, leading to a higher risk of developing a cognitive and/or mood disorders in adulthood [41, 65–67]. We evaluated the effect of pregestational stress (PS) exposure on intensity of anxiety-like behavior by the elevated plus-maze test, a validated behavioral paradigm based on rodent’s preference of closed to open spaces, with latter presenting a significant anxiety-like behavior inducing condition [68]. In this study, we didn’t find significant sex-dependent differences in anxiety-like behavior of juvenile rats, what was expected due to unmature reproductive system [69]. However, female offspring of stressed untreated mothers exhibited decreased anxiety-like behavior that was not present if mothers were treated with MIR. This was changed in adolescence, when females showed decreased anxiety-like behavior if mothers were stressed and treated with MIR. Different results were observed in adolescent male rats, who displayed increased anxiety-like behavior due to pregestational stress without any significant effect of MIR treatment. Changes in anxiety-like behavior, due to maternal stress, were previously described by several research groups [41, 70]. Although there are controversial results regarding effect of chronic pregestational stress on anxiety-like behavior of offspring, possibly due to the different stress paradigm strategies, a great proportion of studies postulate that male offspring whose mothers were submitted to stress before or during pregnancy are more reluctant to enter and spent time in the open arm of the elevated plus maze [71–73]. Effect of perinatal mirtazapine treatment on anxiety-like behavior of offspring, in our study present only in females, may be due to the antagonism that mirtazapine exerts on noradrenergic autoreceptors that in turn enhances the noradrenergic transmission opposing the stress-related response [74]. However, the research group of Sahoo et al. studied the effects of prenatal exposure to mirtazapine on the postnatal development of rats, with animals spent less time in the open arm, albeit sex was not distinguished [75]. Nevertheless, the sex-dependent differences in adaptive behavioral performance to a challenge due to maternal stress may be explained by the influence of individual fetal brain programming, differentially modulating e.g. the responsiveness to a new stimuli, or a hormone arousal during adolescence [76]. Recent studies postulate that the sexual hormones, testosterone and estradiol, have a neuroprotective and anti-inflammatory role on cognitive functions [1, 77], which is a possible ground for further investigation.

### Depressive-like behavior

Modified forced swim test, based on the protocol established by Porsolt [78], has been used as a tool to determine the learned helplessness (rodent despair) and therapeutic efficacy of mirtazapine. However, several recent studies postulate that the immobility in this test may not be a sign of a depressive-like state but rather reflection of the individual ability to cope with an acute stressor, explaining the passive phenotype as a result of animal’s difficulties to adapt to external stimuli [79, 80]. Our results show an increased active behavior of animals from stressed mothers of both sexes regardless of mirtazapine treatment manifested as less time floating and more time trying to actively escape. Even though several studies declared increased immobility time in animals that have experienced stress during their lives, particularly prenatally, [66, 81, 82] there are studies showing possibility of prenatal stress inducing adaptive changes contributing to stress resilience later in life [83–85]. Active behaviors, such as swimming and climbing, in the rat forced swimming test are differentially regulated by serotonergic and noradrenergic systems [86]. Swimming behavior, in this test, is modulated by serotonergic neurotransmission, as Brummelte and colleagues demonstrated a decreased swimming of offspring whose mothers were exposed to high levels of corticosterone while pregnant [87]. Also, Vázquez et al. suggested that increased levels of serotonin in control animals after FST may be responsible for shorter time spent immobile and increased time spent swimming, corroborating an effect of the serotonergic system, which in turn modulates dopaminergic projections associated with the reward system and the coping with stress. In this terms, lower levels of serotonin were linked to a stress-induced anhedonic state, while higher levels promote a non-anhedonic state [66]. Accordingly, higher levels of serotonin may be a plausible explanation to our results.

### Spatial memory

We encountered sex-dependent differences in the Y-maze, a behavioral paradigm set to assess the spatial memory performance building on the natural inclination of rodents to explore new environments. Male offspring from mirtazapine treated mothers showed reduced locomotive activity, indicating an influence of treatment on executive capacity. On the other hand, females seemed to be rather affected by the pregestational stress experience of the mothers, which induced a hyperactivity-like response. Such a result may be reflective of hyperactivity or even ADHD described in children of stressed mothers while pregnant [88, 89]. However, sex differences in reactivity to either pre-gestational stress or perinatal mirtazapine treatment related to potentially hyperactive behavior require further investigation. In children, PS is connected to cognitive, behavioral, and emotional problems such autism and ADHD [90, 91]. The maternal stress, manifesting as increased cortisol and corticotropin-releasing factor (CRF) levels, affects the fetal development by reprogramming the HPA axis, leading to impaired memory and learning due to the long-lasting effects in the hippocampus [92]. Moreover, Deminière et al. had previously described an increased locomotor reactivity to novelty in litters of pre-gestationally stressed mothers, which authors associated with a modified dopaminergic activity in the prefrontal cortex and nucleus accumbens [93]. Experience-dependent plasticity of hippocampal neurons and adult neurogenesis, processes of importance for optimal learning or memory formation and processing, are modulated through epigenetic mechanisms, which induce long-lasting changes determining the ability of individuals to cope with adverse situations [94, 95]. In our study, adolescent females had impaired spatial memory, represented by decreased percentage of spontaneous alternations, as a result of maternal stress as well as mirtazapine treatment. However, this effect was not present in males. Conrad et al. studied the effects of chronic stress on spatial memory performance of adult rats concluding that stress impaired the spatial learning and memory, hippocampi-dependent spatial tasks. Authors also resolved that females seem to be less vulnerable to these hippocampi-dependent memory deficits [51, 96]. Nevertheless, they didn’t study the effects in the offspring. Otherwise, female rats have been linked to more active response in novel environments [43, 97, 98] and, in line with our results, pregestational stress as well antidepressant treatment highlighted this response with effect of mirtazapine being highly visible in animals without pregestational stress exposure. Similar results have been postulated in association with other antidepressant such as fluoxetine, bupropion or citalopram [88, 99]. Nevertheless, our results show a clear influence of pre-gestational stress and antidepressant treatment in the onset of behavioral tasks, which may be driven by the differential strategies used by each sex to cope with adversity. Moreover, we could take into account that we evaluated animals during adolescence, which is known as a time of increased physiological and psychological changes, which make them more vulnerable and unpredictable to external influences [100–102].

### Synaptophysin analysis

Synaptophysin is used as a marker of synaptic plasticity and synaptic nerve terminal density [103]. We analyzed optical density (OD) of synaptophysin, a calcium-binding glycoprotein widely distributed in the presynaptic vesicle membrane, that is required for vesicle fusion and neurotransmitter release, in the CA3, CA4 and DG areas of the hippocampus. Results of synaptophysin density in our study were age-dependent with no changes in juvenile offspring. However, with onset of adolescence we observed prominent effect of sex with synaptophysin OD males remaining intact under all conditions while females being influenced by maternal mirtazapine treatment. This effect was regionally specific as changes were observed only in CA3 and CA4 areas but not in DG. Sex differences can be seen in the neural plasticity at DG-CA3 synapses. In short, mossy fibers evoke larger population spikes in CA3 pyramidal neurons in females during proestrus and estrus relative to males, while mossy fibers in males have stronger synaptic connections to CA3 neurons than females [104]. Limitation of our study is that we didn’t evaluate estrus cycle phase before extraction of the brains. There is very limited research available on maternal antidepressant treatment in association with synaptophysin OD. Fluoxetine, a SSRI, has been proven to impact synaptophysin OD of CA3 in the same sex- and age-dependent manner as seen in our study [44]. Previous studies have encountered a reduced expression of synaptophysin in the hippocampus as whole or in DG, no research is done on other specific regions, at different ages (PP7, PP14, adult) of pre-gestationally stressed pups [105–107], suggesting the involvement of early-life stress in modulation of synaptic plasticity, which could lead to development of neuronal deficits during adulthood [108, 109]. Previous studies suggested a regulatory role of ovarian hormones in the developing brain [108] explaining the differential effect of stress on limbic synaptic plasticity in female rats. However, there are differences between our study compared to studies previously mentioned. Our model comprises of CUS prior to gestation, and we tested the offspring in adolescent age, when the reproductive system of the offspring is not fully developed, so we can’t rule out other stress related changes in later life that were not seen in this study.

## Conclusions

Neurobiology of depression remains mostly unexplained with several theories trying to identify its etiology including the impairment of monoaminergic systems coupled with an altered neural plasticity, dysregulation of the HPA axis or the immunological response. Maternal depression as well as antidepressant treatment thus may lead to a broad spectrum of neurodevelopmental changes in the offspring. Our results suggest mirtazapine induced alterations in maternal behavior and several sex- and age-dependent changes in neurobehavioral development of offspring caused by either or combination of prenatal mirtazapine treatment and chronic pre-gestational stress such as decreased anxiety-like behavior in females, increased anxiety-like behavior in males and impaired spatial memory in females.
